# Editorial: Remodeling Composition and Function of Microbiome by Dietary Strategies - Functional Foods Perspective

**DOI:** 10.3389/fnut.2021.811102

**Published:** 2021-11-30

**Authors:** Silvia Turroni, Alfonso Benítez-Páez

**Affiliations:** ^1^Unit of Microbiome Science and Biotechnology, Department of Pharmacy and Biotechnology, University of Bologna, Bologna, Italy; ^2^Host-Microbe Interactions in Metabolic Health Laboratory, Principe Felipe Research Center (CIPF), Valencia, Spain

**Keywords:** human gut microbiota, functional foods, prebiotics, probiotics, synbiotics, human health, nutrition

Microbes inhabiting the human gastrointestinal tract have been under the spotlight during the last decade, given the multiple associations detected between specific microbiota profiles and health status. Diet is widely recognized as the primary environmental variable shaping the intestinal microbiota in humans. Therefore, the study of diet-microbiota-host interactions deserves special attention to provide clues to several diseases, including cognitive, metabolic, and immune ones. In a similar manner, the investigation of the molecular cross-talk between host cells and microbes in a particular nutritional environment also serves as the foundation for design of innovative therapeutic strategies based on probiotics, prebiotics, and synbiotics. For instance, a recent investigation based on resistant starch suggests that discrete dietary fiber structures can be used to target the production of short-chain fatty acids (1), the major microbiota-derived effector molecules known to have a wide range of action on host health (2). On the other hand, the gut microbiota has been disclosed to modulate the effect of dietary fiber on host health, supporting the notion that there is no one-fits-all diet in the way to seek cost-effective nutritional strategies for health improvement and weight control (3). Anyhow, consensual benefits for human health in microbiota-targeted dietary interventions are still perceived, pointing out, for instance, fermented foods as attenuators of inflammation, and modulators of gut microbiota (4).

The aim of the Frontiers in Nutrition Research Topic (RT) “Remodeling Composition and Function of Microbiome by Dietary Strategies—Functional Foods Perspective” was to assemble clinical and pre-clinical studies deciphering the microbiome-driven effects on human health of innovative functional foods based on probiotics, prebiotics or synbiotics, as well as dietary supplements. We provide an overview of this RT, including five original research articles and two review articles.

## Novel Concepts in Probiotics and Prebiotics

Species belonging to the former *Lactobacillus* genus (5) have a long history of safety and utilization as probiotics for the amelioration of multiple disease conditions in children and adults. Zhao et al. show an updated perspective on the utilization of lactobacilli strains to treat and prevent cardiovascular disease, by dissecting in a species-specific manner the probable molecular mechanisms that explain their efficacy as seen in multiple animal and clinical studies (Zhao et al.). Besides, an intriguing outlook of polyphenols as potential prebiotic compounds is also included in this RT. Rodríguez-Daza et al. provide an updated vision of polyphenols as a wide variety of plant-based bioactive agents that shape the intestinal microbiota via creating new ecological niches by inhibiting pathogens and serving as fermentable compounds by certain gut microbes (Rodriguez-Daza et al.). The concept of polyphenol-associated enzymes (PAZymes) is introduced and defined as a potential biomarker to distinguish bacteria that can utilize these compounds to improve their fitness and persistence in intestinal niches, while also prompting host health benefits.

## Hidden Potential of Singular Ingredients of Plant and Insect Origin

Protein-based diets and supplements are often adopted for weight loss goals and concomitantly produce drastic effects on host metabolism that may be mediated by the gut microbiota. Consequently, Wang et al. present a 12-week animal trial upon a high-fat diet (HFD), and supplementation with a *Phaseolus vulgaris* extract (PVE) enriched with an α-amylase inhibitor. This pre-clinical study showed that PVE exerted a protective effect on the impairment of glucose metabolism and weight gain, both intrinsic characteristics of HFD regimes. Moreover, PVE attenuated the circulating pro-inflammatory state observed in mice resulting from HFD exposure. The authors argued that the α-amylase inhibitor would favor the bioavailability of starch and glycogen in distal portions of the host intestine, thus providing more polysaccharides for fermentation by the gut microbiota. Therefore, the beneficial effects could be linked to microbial species boosted by PVE (Wang et al.). On the other hand, a yucca schidigera extract (YSE) combined with a *Candida utilis* (CU) strain is explored to improve piglet growth and reduce post-weaning diarrhea and mortality. Such a combined strategy could have important implications in the animal husbandry industry, as the authors found that the synergistic effect of YSE+CU on piglet survival after weaning likely underlies their improved multi-organ function and the boost of natural anti-microbial response in their gastrointestinal tract (Yang et al.).

Chitosan oligosaccharides (COS), resulting from insect chitin processing, were tested in a mouse model with HFD-induced obesity. The pre-clinical trial conducted by Wang et al. showed that COS exert potential prebiotic effects by boosting the abundance of recognized beneficial bacteria, such as *Akkermansia muciniphila* and *Bacteroides uniforms*. Furthermore, COS appear to protect against excessive weight gain and dyslipidemia characteristic of obesogenic diets. Concomitantly, COS are likely to strengthen the gut barrier and attenuate pro-inflammatory signaling in the gastrointestinal tract (Wang et al.). Finally, the potential prebiotic effect of Korean plant-based *saengshik* on the gut microbiota was examined in a clinical trial with 66 healthy subjects. The results described by Shin et al. indicate that the gut microbiota response to *saengshik* is quite heterogeneous, but with a recognized boost of health-associated microbes such as *Faecalibacterium* species, and attenuation of pro-inflammatory species, depending on individual dietary patterns (Shin et al.).

## Rational Design of Multi-Strain Probiotic Products

The study completed by Wang et al. represents an engaging disease-targeted probiotic design and development with a particular application on chronic kidney disease (CKD). Three strains characterized to attenuate indole production by other gut microbes (*Lactobacillus acidophilus, Bifidobacterium longum*, and *Bifidobacterium bifidum*), which is a precursor of cytotoxic indoxyl sulfate and accumulates in the kidneys of patients with poor renal function, were combined and orally administrated in a CDK mouse model, exhibiting anti-inflammatory effects and maintenance of glomerular structures upon sustained multi-strain product intake. In an ambitious proof-of-concept of its clinical application, Wang et al. also show their multi-strain product retards the progression of renal failure in CKD patients by declining the estimated glomerular filtration rate (eGFR) during a 6-month trial (Wang et al.).

All in all, we firmly believe that the contributions gathered in this RT represent a clear advance in the design of novel strategies to remodel the human gut microbiome, providing a fresh perspective and paving the way for future studies and clinical applications of the functional foods introduced here.

## Author Contributions

All authors listed have made a substantial, direct, and intellectual contribution to the work and approved it for publication.

## Conflict of Interest

The authors declare that the research was conducted in the absence of any commercial or financial relationships that could be construed as a potential conflict of interest.

## Publisher's Note

All claims expressed in this article are solely those of the authors and do not necessarily represent those of their affiliated organizations, or those of the publisher, the editors and the reviewers. Any product that may be evaluated in this article, or claim that may be made by its manufacturer, is not guaranteed or endorsed by the publisher.
